# Supplementary material for: 2'-O-ribose methylation of transfer RNA promotes recovery from oxidative stress in Saccharomyces cerevisiae
Source: PLoS One. 2020 Feb 13;15(2):e0229103. doi: 10.1371/journal.pone.0229103 (PMC7018073; doi:10.1371/journal.pone.0229103)
Supplement: S1 Fig — (DOCX) [file pone.0229103.s001.docx]

**
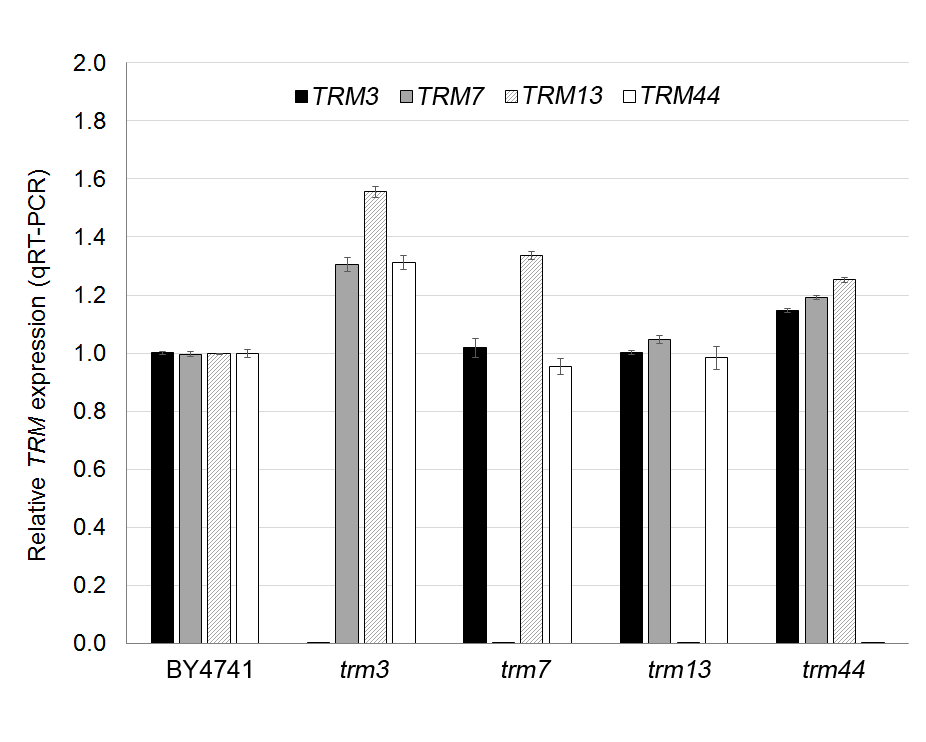
**

**S1 Fig. *TRM* expression in wildtype and mutant strains**. Quantitative real-time reverse transcription-based PCR (qRT-PCR) was used to assess the expression of *TRM* gene transcripts in BY4741 and *trm* mutant strains. Gene expression assays (TaqMan®) determined *TRM*-specific expression in cDNAs generated from each mutant strain relative to cDNA generated from BY4741 using the delta-delta cycle threshold (2^-ΔΔCt^) method, with actin serving as the internal control.
