# Supplementary material for: 2'-O-ribose methylation of transfer RNA promotes recovery from oxidative stress in Saccharomyces cerevisiae
Source: PLoS One. 2020 Feb 13;15(2):e0229103. doi: 10.1371/journal.pone.0229103 (PMC7018073; doi:10.1371/journal.pone.0229103)
Supplement: S2 Fig — (DOCX) [file pone.0229103.s002.docx]

**
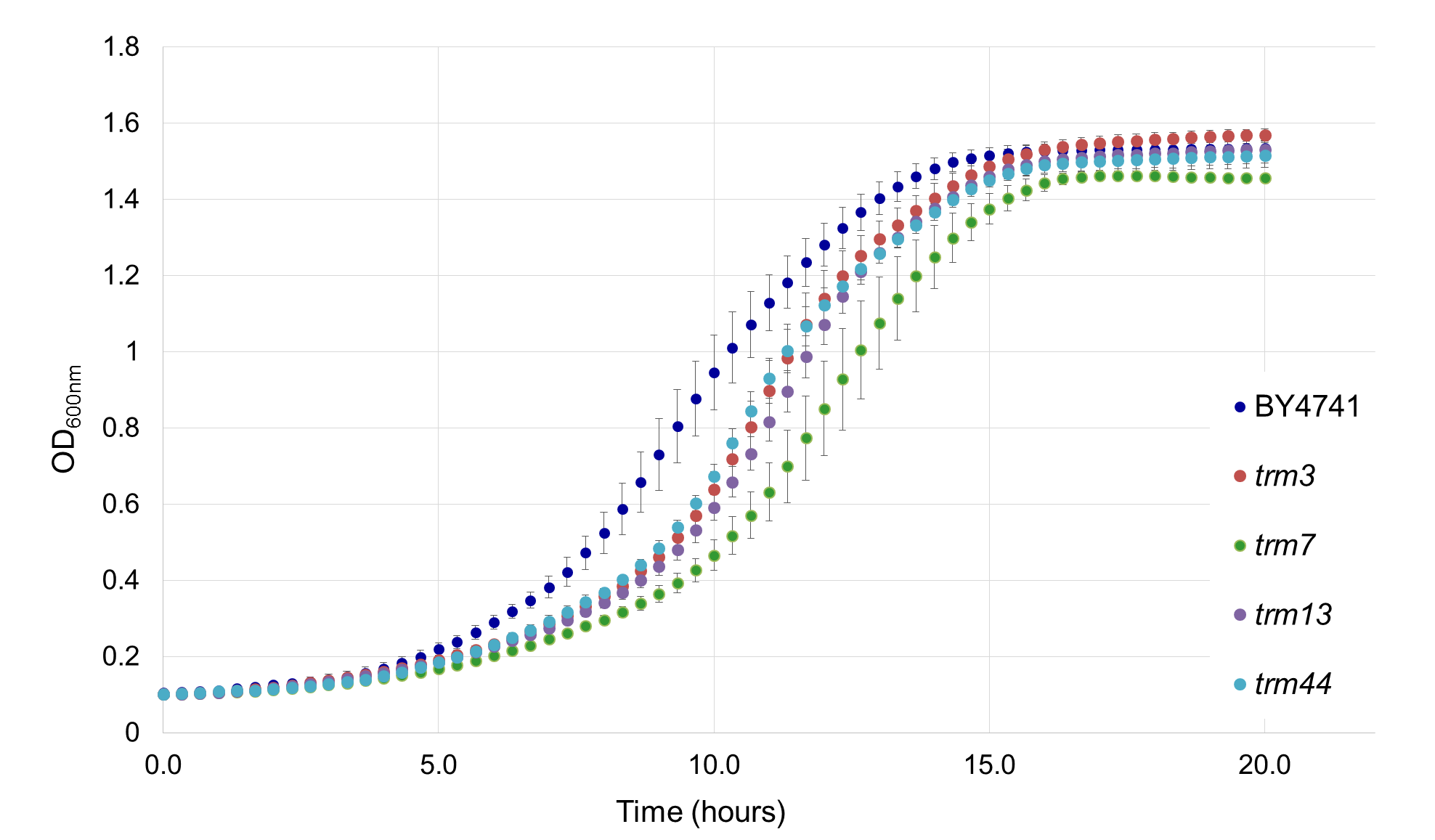
**

**S2 Fig. Unchallenged growth of 2’-O-ribose trm mutants.** Wild type (BY4741) and *trm* mutant strains were grown from a single colony overnight in liquid YPD supplemented with G418 (i.e., deletion mutants only), and then serially diluted to 7 x 10^-3^. Growth was monitored by measuring the optical density of the cultures based on their absorbance at 600 nm every twenty-minutes until the cultures reached a stationary phase of growth (error bars are + standard deviation of the mean, n=5).
