# Supplementary material for: 2'-O-ribose methylation of transfer RNA promotes recovery from oxidative stress in Saccharomyces cerevisiae
Source: PLoS One. 2020 Feb 13;15(2):e0229103. doi: 10.1371/journal.pone.0229103 (PMC7018073; doi:10.1371/journal.pone.0229103)
Supplement: S1 Table — (DOCX) [file pone.0229103.s003.docx]

| **PTM** | **BY4741_UT** | ***trm3*_UT** | ***trm7*_UT** | ***trm13*_UT** | ***trm44*_UT** |
| --- | --- | --- | --- | --- | --- |
| **A** | 23.479  ± 1.629 | 22.538  ± 1.987 | 20.714  ± 0.443 | 23.813  ± 4.087 | 26.057  ± 3.977 |
| **ac^4^C, f^5^Cm** | 0.512  ± 0.012 | 0.520  ± 0.013 | 0.548  ± 0.012 | 0.502  ± 0.057 | 0.487  ± 0.048 |
| **ac^4^Cm** |  | 0.003  ± 0.001 | 0.003  ± 0.002 |  |  |
| **C** | 20.670  ± 1.949 | 21.390  ± 1.344 | 21.414  ± 0.030 | 21.993  ± 0.234 | 19.990  ± 0.797 |
| **cmnm^5^U** |  | 0.006  ± 0.001 | 0.008  ± 0.007 |  |  |
| **D** | 1.494  ± 0.219 | 2.405  ± 0.897 | 1.588  ± 0.296 | 2.831  ± 0.516 | 1.634  ± 0.368 |
| **G** | 30.849  ± 2.621 | 30.360  ± 1.913 | 32.161  ± 0.201 | 31.082  ± 3.178 | 27.583  ± 3.851 |
| **I** | 0.068  ± 0.010 | 0.108  ± 0.030 | 0.070  ± 0.015 | 0.171  ± 0.009 | 0.069  ± 0.019 |
| **i^6^A** |  | 0.027  ± 0.014 | 0.007  ± 0.003 | 0.049  ± 0.056 | 0.020  ± 0.020 |
| **Am** | 0.248  ± 0.036 | 0.301  ± 0.023 | 0.204  ± 0.052 | 0.406  ± 0.082 | 0.265  ± 0.043 |
| **Gm** | 0.754  ± 0.138 | 0.931  ± 0.200 | 0.712  ± 0.134 | 1.199  ± 0.134 | 0.709  ± 0.162 |
| **m^1^Gm, m^2^_2_G, m^2^Gm** | 0.228  ± 0.031 | 0.327  ± 0.064 | 0.215  ± 0.053 | 0.453  ± 0.025 | 0.220  ± 0.042 |
| **m^1^I, Im** |  |  | 0.001  ± 0.001 |  |  |
| **Cm** | 0.705  ± 0.069 | 0.941  ± 0.178 | 0.624  ± 0.067 | 1.146  ± 0.038 | 0.652  ± 0.105 |
| **m^6^Am, m^1^Am, m^6^_2_A** | 0.030  ± 0.007 | 0.032  ± 0.030 | 0.013  ± 0.003 |  |  |
| **mcm^5^s^2^U** |  | 0.013  ± 0.014 |  |  |  |
| **mcm^5^U** |  | 0.029  ± 0.014 | 0.014  ± 0.010 | 0.021  ± 0.016 | 0.011  ± 0.009 |
| **ncm^5^U** |  |  | 0.026  ± 0.017 | 0.031  ± 0.012 |  |
| **Um** | 0.740  ± 0.141 | 0.976  ± 0.285 | 0.668  ± 0.068 | 1.095  ± 0.091 | 0.678  ± 0.109 |
| **Y,U** | 25.002  ± 2.189 | 25.712  ± 1.413 | 25.710  ± 0.674 | 23.112  ± 1.605 | 26.371  ± 1.525 |
| **yW** | 0.017  ± 0.002 |  |  |  |  |

**S1 Table. Post-transcriptional RNA modifications, unchallenged.** The relative abundance of post-transcriptional RNA modifications (PTMs) in unchallenged wildtype (BY4741) and *trm* mutants (i.e., UT: untreated). The average numerical values of each PTM were calculated using the Abundance versus Proxy method (AvP) from three independent samples, as described in the methods section. The standard deviation associated with these values is shown in parenthesis. The cyan blue shade indicates a down-regulation from the BY4741 UT, while the dark blue indicates an up-regulation with a 95% confidence that the values are significantly different (Student t-test, n=3, P<0.05).  PTM values below detectable levels are indicated by the empty cells; red shaded cells indicate PTMs that were detectable in some strains but not observed in the BY4741 UT (assigned a value of zero).
