# Supplementary material for: 2'-O-ribose methylation of transfer RNA promotes recovery from oxidative stress in Saccharomyces cerevisiae
Source: PLoS One. 2020 Feb 13;15(2):e0229103. doi: 10.1371/journal.pone.0229103 (PMC7018073; doi:10.1371/journal.pone.0229103)
Supplement: S2 Table — (DOCX) [file pone.0229103.s004.docx]

| **PTM** | **BY4741_UT** | **BY4741_T** | ***trm3*_T** | ***trm7*_T** | ***trm13*_T** | ***trm44*_T** |
| --- | --- | --- | --- | --- | --- | --- |
| **A** | 23.479  ± 1.629 | 25.159  ± 2.050 | 25.002  ± 1.443 | 27.226  ± 3.224 | 25.551  ± 1.372 | 26.724  ± 2.360 |
| **ac^4^C, f^5^Cm** | 0.512  ± 0.012 | 0.413  ± 0.049 | 0.317  ± 0.082 | 0.165  ± 0.082 | 0.437  ± 0.062 | 0.162  ± 0.029 |
| **ac^4^Cm** |  |  |  |  |  |  |
| **C** | 20.670  ± 1.949 | 22.430  ± 1.503 | 21.897  ± 2.424 | 18.385  ± 1.178 | 22.123  ± 1.118 | 19.703  ± 1.401 |
| **cmnm^5^U** |  |  |  |  |  |  |
| **D** | 1.494  ± 0.219 | 2.600  ± 0.738 | 2.437  ± 0.965 | 0.558  ± 0.225 | 2.829  ± 0.099 | 2.084  ± 0.696 |
| **G** | 30.849  ± 2.621 | 31.727  ± 4.200 | 31.954  ± 4.122 | 31.806  ± 4.798 | 30.283  ± 4.252 | 32.223  ± 3.918 |
| **ho^5^U** |  |  |  | 0.026  ± 0.015 |  |  |
| **I** | 0.068  ± 0.010 | 0.079  ± 0.075 | 0.259  ± 0.364 |  | 0.096  ± 0.071 |  |
| **i^6^A** |  |  |  |  |  |  |
| **Am** | 0.248  ± 0.036 | 0.231  ± 0.179 | 0.135  ± 0.129 |  | 0.333  ± 0.136 | 0.089  ± 0.115 |
| **Gm** | 0.754  ± 0.138 | 1.059  ± 0.208 | 0.729  ± 0.254 | 0.135  ± 0.051 | 1.177  ± 0.070 | 0.666  ± 0.258 |
| **m^1^Gm, m^2^_2_G, m^2^Gm** | 0.228  ± 0.031 | 0.438  ± 0.201 | 0.305  ± 0.217 | 0.0047  ± 0.002 | 0.490  ± 0.016 | 0.123  ± 0.122 |
| **m^1^I, Im** |  |  |  |  |  |  |
| **Cm** | 0.705  ± 0.069 | 1.198  ± 0.247 | 1.045  ± 0.437 | 0.0775  ± 0.048 | 1.202  ± 0.029 | 0.744  ± 0.369 |
| **m^6^Am, m^1^Am, m^6^_2_A** | 0.030  ± 0.007 |  |  |  |  |  |
| **mcm^5^s^2^U** |  |  |  |  |  |  |
| **mcm^5^U** |  |  |  |  |  |  |
| **ncm^5^U** |  |  |  |  |  |  |
| **Um** | 0.740  ± 0.141 | 0.919  ± 0.260 | 0.828  ± 0.371 | 0.124  ± 0.085 | 1.064  ± 0.061 | 0.586  ± 0.393 |
| **Y,U** | 25.002  ± 2.189 | 20.684  ± 0.856 | 21.147  ± 1.218 | 22.583  ± 1.806 | 22.044  ± 1.839 | 21.349  ± 0.185 |
| **yW** | 0.017  ± 0.002 |  |  |  |  |  |

**S2 Table. Hydrogen peroxide-induced post-transcriptional modifications.** The figure legend for the supplemental table 3 (S3 Table) also applies to this table, only here T represents hydrogen peroxide treated. Values for the UT wildtype BY4741 strain are shown again to assist the reader.
