# Supplementary material for: 2'-O-ribose methylation of transfer RNA promotes recovery from oxidative stress in Saccharomyces cerevisiae
Source: PLoS One. 2020 Feb 13;15(2):e0229103. doi: 10.1371/journal.pone.0229103 (PMC7018073; doi:10.1371/journal.pone.0229103)
Supplement: S3 Table — (DOCX) [file pone.0229103.s005.docx]

| **Transcript ID** | **Gene name** | **Locus** |
| --- | --- | --- |
| YNR016C | *ACC1* | chrXIV(-):654673-661374 |
| YCL025C | *AGP1* | chrIII(-):76018-77919 |
| YJL075C | *APQ13* | chrX(-):298460-298876 |
| YNR019W | *ARE2* | chrXIV(+):665339-667267 |
| YLR062C | *BUD28* | chrXII(-):263577-263954 |
| YOL063C | *CRT10* | chrXV(-):207392-210265 |
| YDR051C | *DET1* | chrIV(-):557056-558060 |
| YOR223W | *DSC3* | chrXV(+):759782-760660 |
| YGL054C | *ERV14* | chrVII(-):400871-401287 |
| YOR384W | *FRE5* | chrXV(+):1061564-1063648 |
| YDR508C | *GNP1* | chrIV(-):1466453-1468444 |
| YJR140C | *HIR3* | chrX(-):690750-695696 |
| YEL069C | *HXT13* | chrV(-):21537-23231 |
| YDL245C | *HXT15* | chrIV(-):11657-13360 |
| YJR158W | *HXT16* | chrX(+):732440-734143 |
| YMR011W | *HXT2* | chrXIII(+):288079-289704 |
| YHR092C | *HXT4* | chrVIII(-):287081-288811 |
| YGR249W | *MGA1* | chrVII(+):988049-989419 |
| YPL058C | *PDR12* | chrXVI(-):445842-450377 |
| YHR074W | *QNS1* | chrVIII(+):246193-248337 |
| YBR150C | *TBS1* | chrII(-):541209-544493 |
| YOR376W | Uncharacterized ORF | chrXV(+):1043191-1043559 |
| YOL128C | *YGK3* | chrXV(-):78352-79479 |
| YNL160W | *YGP1* | chrXIV(+):336545-337609 |

**S3 Table. UUC-enriched open reading frames (ORFs).** CUT-identified *S. cerevisiae* ORFs containing a triplet UUCUUCUUC codon run.
